# Media framing of traditional Chinese medicine and its public health implications: a cross-national panel analysis of news coverage in the US, UK, and Europe (2010–2024)

**DOI:** 10.3389/fpubh.2026.1826131

**Published:** 2026-06-12

**Authors:** Zhen-yu Gu, Yan Chen, A-jian Zhou

**Affiliations:** 1 The International Education College, Nanjing University of Chinese Medicine, Nanjing, China; 2 Beijing Jiayi Language Service Co., Ltd., Beijing, China

**Keywords:** COVID-19, cross-national comparison, health communication, media framing, panel data, public health policy, traditional Chinese medicine

## Abstract

**Introduction:**

Western media framing of traditional Chinese medicine (TCM) shapes public health beliefs and policy attitudes across national contexts, yet systematic cross-national evidence remains limited.

**Methods:**

This study analyzed TCM-related news coverage across six Western countries, the United States, the United Kingdom, Germany, France, Spain, and Italy, from 2010 to 2024. Using a unified four-category coding framework applied to a multilingual corpus of elite print and online outlets, we constructed a balanced panel of 360 country-quarter observations and employed two-way fixed effects regression models to examine cross-national variation in frame distribution and sentiment orientation.

**Results:**

Risk/safety frames dominated in the United States (38.4%) and the United Kingdom (35.1%), whereas scientific/efficacy frames prevailed in Germany (41.2%) and France (37.8%). The COVID-19 pandemic improved overall sentiment (*β* = 0.14, *p* < 0.001) and intensified risk framing (*β* = 0.11, *p* < 0.01), with risk amplification concentrated in NHS-type healthcare systems. WHO ICD-11 inclusion of TCM increased policy frame ratios (*β* = 0.08, *p* < 0.01) without improving evaluative tone, while domestic TCM legislative developments improved sentiment and policy framing but did not affect risk framing.

**Discussion:**

These findings suggest that national regulatory contexts and major public health shocks are consistent correlates of TCM media framing patterns. However, the small number of country clusters and uneven article density, especially in Southern European countries, limit inferential precision and preclude strong causal claims. The findings support country-specific rather than uniform international public health communication strategies for TCM, pending audience-level confirmation.

## 1 Introduction

Traditional Chinese Medicine has gained unprecedented institutional visibility in Western healthcare discourse over the past two decades. The WHO’s formal adoption of TCM diagnostic categories into the International Classification of Diseases (ICD-11) at the 72nd World Health Assembly in May 2019 (Q2 2019) (1), combined with heightened global demand for complementary health options during the COVID-19 pandemic (2, 3), has accelerated TCM’s entry into mainstream public health debate across Western societies. Yet public receptivity to TCM remains deeply uneven across national contexts. Utilization rates, regulatory frameworks, and policy attitudes toward TCM modalities such as acupuncture and herbal medicine differ substantially between the United States, the United Kingdom, and Continental European countries, suggesting that factors beyond clinical evidence alone shape cross-national variation in TCM acceptance (4–6).

News media occupy a structurally central role in this dynamic. For most Western publics, news coverage constitutes the primary source of information about therapeutic systems outside the biomedical mainstream. The frames through which journalists present TCM—emphasizing clinical evidence, potential harms, cultural origins, or regulatory status—shape the cognitive environment within which individuals form beliefs about TCM’s credibility and appropriateness as a health-seeking option (7, 8). A media environment dominated by risk frames may suppress public willingness to consider TCM even where evidence supports its efficacy, while policy and scientific frames may facilitate greater openness to integrative medicine approaches. Media framing is therefore not merely a reflection of public attitudes toward TCM but an active force in their cross-national differentiation (9).

Despite the theoretical importance of media framing to cross-national TCM receptivity, the existing literature remains fragmented. Existing studies are predominantly single-country in design, limited to short observation windows, and rely on qualitative methods whose cross-linguistic replicability is difficult to assess. Recent work has begun to address this gap: Tang et al. (10) applied uncertainty avoidance theory to a content analysis of TCM news coverage across seven countries, and Pan (11) used corpus-assisted discourse analysis to examine TCM representation in international academic literature. These contributions represent concurrent and complementary work; however, they rely on cross-sectional rather than longitudinal panel designs, do not employ two-way fixed effects regression capable of separating country-level structural factors from time-varying shocks, and do not apply a common multi-language coding protocol across six national media systems simultaneously. No published study has applied a unified coding framework within a balanced multi-country panel spanning 15 years and used two-way fixed effects regression to decompose cross-national heterogeneity in framing and sentiment, leaving it unclear whether nationally observed framing differences reflect genuine structural heterogeneity or methodological inconsistency across research designs (12).

This study addresses this gap through a cross-national panel data analysis of TCM-related news coverage across six Western countries—the United States, the United Kingdom, Germany, France, Spain, and Italy—from January 2010 to December 2024. Applying a unified content coding framework to a systematically constructed multilingual media corpus and employing two-way fixed effects panel regression models, this study addresses three research questions: (RQ1) What patterns of cross-national variation in TCM media framing are observable across the six countries, and how have these patterns evolved in response to major public health events? (RQ2) To what extent do structural factors—including healthcare system type, national TCM regulatory status, and major public health shocks—account for observed cross-national framing differences? (RQ3) What are the implications of documented framing heterogeneity for public health communication strategy and integrative medicine policy in Western contexts?

This study contributes to the literature in two respects. Methodologically, it introduces panel data econometric modeling to the health communication research on TCM, enabling more rigorous decomposition of cross-national variance than case-comparative designs permit. Substantively, it generates descriptive cross-national evidence that may inform hypotheses about the design of differentiated public health communication strategies for TCM across Western media environments, though direct testing of such strategies requires audience-level data beyond the scope of the present study.

## 2 Literature review

### 2.1 Media framing theory and health communication

Framing theory, as foundational work by Entman (7) conceptualizes it, holds that media do not merely transmit information but actively select, emphasize, and organize elements of perceived reality in ways that promote particular problem definitions, causal interpretations, moral evaluations, and treatment recommendations. In the domain of health communication, this theoretical tradition has proven particularly generative. Scheufele and Tewksbury (8) elaboration of framing as a process of audience cognition construction demonstrated that health frames systematically shape the criteria by which individuals evaluate health-related claims, assign credibility to therapeutic options, and form attitudes toward health policy. Subsequent empirical work has refined the typology of health-relevant frames into several analytically distinct categories that recur across substantive domains (13).

The scientific/efficacy frame foregrounds empirical evidence, clinical trial data, and expert medical consensus as the primary evaluative criteria for health claims, privileging biomedical epistemology and treating measurable therapeutic outcomes as the legitimate currency of health discourse. The risk/safety frame, by contrast, centers on potential harms, adverse effects, contraindications, and regulatory failures, activating audience risk perception and often triggering precautionary responses in health-seeking behavior. The cultural/political frame contextualizes health phenomena within broader identity, geopolitical, or ideological narratives, positioning therapeutic practices as expressions of national or civilizational values rather than as purely technical interventions. The policy frame focuses on institutional and regulatory dimensions, foregrounding questions of coverage, legalization, standardization, and system integration. These four frame types are not mutually exclusive in practice; a single article may deploy multiple frames simultaneously, and the relative salience of each frame constitutes the primary analytical object of content-analytic research in health communication.

The relationship between media framing and public health outcomes has been extensively documented. Gollust et al. (14) demonstrated via randomized experiment that health news framing significantly predicted public support for health policies, even after controlling for prior attitudes and political predispositions. Slater and Rouner (15) showed that narrative health frames, compared to statistical or evidential frames, produced stronger attitude change under conditions of high audience involvement. These findings collectively establish media framing not merely as a descriptive feature of health journalism but as a consequential determinant of the public health information environment (9, 13, 16).

### 2.2 Western media representations of TCM: a review of existing research

Empirical research on Western news media coverage of TCM has grown steadily since the early 2000s, though it remains methodologically uneven and geographically fragmented (17, 18). The preponderance of existing studies adopts a single-country design with limited time horizons (10–12), constraining the inferential scope of their findings.

Within the United States context, Weeks and Strudsholm (19) scoping review of CAM and mass media coverage found that risk and safety themes—including regulatory concerns and evidence standards for herbal products such as FDA oversight requirements—dominated how TCM-related content was framed across mainstream newspaper coverage. Similarly, Iyengar (20) documented a pattern of episodic rather than thematic framing in political news coverage, a dynamic subsequently identified in CAM-related journalism, whereby individual adverse event reports received disproportionate prominence relative to systematic efficacy evidence, creating a media environment skewed toward risk amplification. More recent work examining online news coverage in the United States has noted a partial softening of risk-dominant framing in the aftermath of the opioid crisis (21), as media narratives increasingly positioned acupuncture and related TCM modalities as potential components of non-pharmacological pain management strategies.

In the United Kingdom, research on TCM media representation has frequently intersected with the broader debate over complementary and alternative medicine regulation following the Pittilo Report of 2008 (22), which recommended statutory regulation of several CAM professions including practitioners of traditional Chinese medicine. Analyses of broadsheet coverage in this period found that scientific/efficacy frames and risk frames coexisted in relative tension (9), with outlets such as The Guardian consistently subjecting TCM efficacy claims to scrutiny based on Cochrane review standards while simultaneously covering regulatory integration debates in a more neutral policy frame. BBC online coverage has been characterized by a notable reliance on authoritative medical source citation, reinforcing biomedical gatekeeping norms that tend to disadvantage TCM claims lacking randomized controlled trial support (13).

Comparative research within Continental Europe remains sparse. German-language studies have noted that TCM coverage in outlets such as Der Spiegel tends to be more heavily weighted toward scientific/efficacy frames than risk frames, a pattern attributed to Germany’s historically accommodating regulatory tradition toward naturopathic medicine and the professional legitimacy accorded to Heilpraktiker practitioners within the German healthcare system. French scholarship on TCM media has been even more limited, though available analyses suggest that Le Monde and related quality press outlets situate TCM within cultural and diplomatic frames more frequently than their Anglo-American counterparts, particularly in periods of elevated bilateral engagement between France and China. Research on Southern European TCM media representation is virtually absent from the English-language literature, representing a significant empirical lacuna that the present study partially addresses.

Across these national studies, three methodological limitations recur. First, the majority of studies examine coverage spanning 5 years or fewer, precluding analysis of long-run trend dynamics and precluding the use of longitudinal statistical methods capable of distinguishing structural patterns from transient fluctuations. Second, most studies rely on qualitative or semi-quantitative content analysis procedures with limited attention to inter-rater reliability, reducing confidence in the replicability of frame classification decisions. Third, and most importantly for the purposes of the present study, no existing published research has attempted a systematic cross-national comparison of TCM media framing using a unified analytical framework and a common coding protocol applied across multiple countries simultaneously, leaving the question of whether observed national differences constitute genuine heterogeneity or artifacts of methodological inconsistency fundamentally unresolved.

### 2.3 Media coverage, public health behavior, and policy attitudes

The relationship between media framing and public health behavior is theorized within multiple complementary frameworks. The Health Belief Model, originally developed by Rosenstock (23) and subsequently elaborated by Janz and Becker (24) identifies perceived susceptibility, perceived severity, perceived benefits, and perceived barriers as the primary cognitive determinants of health-related behavior. Media framing operates on this model principally through its influence on perceived benefits and perceived barriers: risk-dominant media frames elevate the perceived barriers associated with TCM utilization by foregrounding safety concerns and regulatory uncertainty, while scientific/efficacy frames lower perceived barriers by legitimizing TCM modalities through the currency of biomedical evidence. The net effect on health-seeking behavior is therefore a function of the relative balance of frame types in the ambient media environment, a balance that, as documented in Section 2.2, varies substantially across the national contexts examined in the present study (25, 26).

Agenda-setting theory offers a complementary explanatory mechanism. McCombs and Shaw’s foundational work (27) demonstrated that the frequency and prominence with which media cover particular issues shapes the salience of those issues in public cognition, and subsequent second-level agenda-setting research by McCombs et al. (28) extended this logic to show that media also influence which attributes of issues are considered important. Applied to TCM coverage, agenda-setting dynamics predict that sustained increases in media attention, such as those observed during the COVID-19 pandemic across all six countries in the present sample, elevate TCM’s overall salience in public health cognition regardless of the frame through which coverage is delivered (29). However, the attribute agenda-setting mechanism suggests that the dominant frame type in that elevated coverage simultaneously shapes which dimensions of TCM—its therapeutic potential, its safety risks, or its policy status—become most cognitively accessible to public audiences.

The link between media framing and health policy attitudes has been examined most directly in research on vaccine hesitancy, mental health stigma, and obesity policy (16, 30, 31), where risk-dominant framing has been consistently associated with reduced policy support and increased individual blame attributions. Brodie and colleagues demonstrated that entertainment media can serve as an effective channel for communicating health information to the public, with audience exposure associated with measurable gains in health knowledge (30), while Gollust et al. (31) showed that frame exposure effects on health policy attitudes were moderated by prior health knowledge and ideological predispositions. Translating these findings to the TCM context suggests that the risk-dominant media environments of the United States and United Kingdom may systematically depress public support for integrative medicine policy initiatives, while the more scientifically receptive media environments of Germany and France may create more permissive conditions for policy innovation in this domain.

### 2.4 Theoretical framework for cross-national comparison

The cross-national comparative design of this study requires a theoretical framework capable of explaining why structurally similar media systems might nonetheless produce systematically different TCM framing patterns. Two complementary institutional frameworks are drawn upon to address this requirement.

The first is Böhm et al. (32) typology of healthcare system organization, which distinguishes National Health Service systems financed through general taxation with universal coverage and predominantly public provision, as exemplified by the United Kingdom; social health insurance systems financed through employment-based contributions with pluralistic provision, as exemplified by Germany, France, Spain, and Italy; and market-oriented systems characterized by private insurance dominance and limited universal coverage, as exemplified by the United States. This typology is relevant to media framing because healthcare system type shapes the institutional context within which medical claims are evaluated and legitimized. In NHS-type systems, cost-effectiveness and evidence-based gatekeeping norms are institutionally paramount, creating an environment in which unvalidated therapeutic claims—including many TCM modalities—are systematically subjected to heightened scrutiny. In social health insurance systems, the pluralistic provider structure and historically greater accommodation of CAM reimbursement create institutional conditions more conducive to policy-frame discourse around TCM integration. In market-oriented systems, the commercial logic of the healthcare sector interacts with consumer health information demand in ways that simultaneously amplify both promotional and risk-amplifying media framings of novel therapeutic options.

It should be noted that the application of Böhm et al.’s typology to the present six-country sample requires a classification decision with respect to Spain and Italy. While Germany and France represent canonical Bismarckian social health insurance systems financed through employment-based contributions, Spain and Italy underwent structural transitions in the late 1970s and 1980s—establishing the Sistema Nacional de Salud (1986) and Servizio Sanitario Nazionale (1978) respectively—that shifted their financing base toward general taxation and their coverage logic toward universalism. Comparative health systems scholars have variously classified these systems as a distinct “Southern European” sub-type [Ferrera, (33)] or as Beveridgean NHS variants that differ from the German-French social insurance model in institutional logic despite sharing the label of “continental” systems. In the primary analysis, we adopt a four-category extension of Böhm et al.’s typology that distinguishes Spain and Italy as a Southern European NHS sub-type from the Bismarckian social health insurance core of Germany and France. The post-1978/1986 transitions of the Italian Servizio Sanitario Nazionale and the Spanish Sistema Nacional de Salud toward tax-based financing and universal coverage produce institutional logics meaningfully distinct from the employment-based, fund-mediated provision that continues to characterize the German-French model; treating these systems as a single “social insurance” category obscures these institutional differences and risks attributing to one financing logic patterns that may instead reflect another. We retain Böhm et al. (32) as the underlying conceptual scaffold but extend it to four operational categories—Northern NHS (United Kingdom), Bismarckian Social Insurance (Germany, France), Southern European NHS (Spain, Italy), and Market-Oriented (United States, reference)—to better preserve the institutional heterogeneity within the “continental” group. We report the original three-category specification as a sensitivity analysis in Section 4.4 and Table 1, Panel E.

**Table 1:** Robustness checks.

Panel A: alternative time control specifications (key coefficients across three models). Dependent variable: sentiment index			
Variable	Country FE + country trend (primary)	Year FE	Quarter FE
COVID-19	0.13*** (0.03)	0.12*** (0.03)	—
ICD-11 inclusion	0.04 (0.03)	0.05^†^ (0.03)	0.04 (0.03)
TCM legislative status	0.09** (0.03)	0.08** (0.03)	0.09** (0.03)
NHS system (RE)	−0.10^*,‡^ (0.05)	−0.11^*,‡^ (0.05)	−0.11^*,‡^ (0.05)
Within *R*^2^	0.39	0.38	0.41
Observations	360	360	360

Panel B: placebo test results (COVID-19 and ICD-11 dummies, risk frame ratio)			
Placebo assignment quarter	Coefficient	*p*-value	Interpretation
Q1 2016	0.07	0.08	Borderline—noted as boundary case
All other 499 iterations (median)	0.01	>0.10	Consistent with null
Baseline estimate (true COVID-19)	0.11	<0.01	Main finding confirmed
ICD-11 placebo (risk frame ratio)			
All 500 iterations (median)	0.01	>0.10	Consistent with null
Baseline estimate (true ICD-11)	0.03	0.28	Not significant; placebo indistinguishable from baseline

Panel C: leave-one-out sensitivity analysis (RE, sentiment index)				
Country excluded	NHS coef.	Social insurance Coef.	COVID-19 coef.	ICD-11 coef.
None (full sample)	−0.11^*,‡^	0.03	0.13***	0.04
United States	−0.10^**,‡^	0.03	0.13***	0.04
United Kingdom	—	0.04	0.13***	0.04
Germany	−0.12^**,‡^	0.02	0.14***	0.05
France	−0.11^*,‡^	0.04	0.13***	0.04
Spain	−0.11^*,‡^	0.03	0.12***	0.04
Italy	−0.10^**,‡^	0.03	0.13***	0.03

Panel D: alternative dependent variable specification (binary high-sentiment indicator)		
Variable	Baseline (country FE + country trend, continuous)	Alternative (country FE + country trend, binary)
NHS system	−0.11^*,‡^ (0.05)	−0.09^**,‡^ (0.03)
Social insurance system	0.03 (0.05)	0.02 (0.04)
TCM legislative status	0.09** (0.03)	0.08** (0.03)
COVID-19	0.14*** (0.03)	0.12*** (0.03)
ICD-11 inclusion	0.04 (0.03)	0.03 (0.03)
Within *R*^2^	0.41	0.38
Observations	360	360

Panel E: three-category typology sensitivity check (sentiment index, RE specification)		
Variable	Sensitivity check (3-category, original Böhm)	Primary typology (4-category)
NHS/northern NHS (UK)	−0.11^*,‡^ (0.05)	−0.13^*,‡^ (0.06)
Social insurance/Bismarckian (DE, FR)	0.03 (0.05)	0.08* (0.04)
Southern European NHS (ES, IT)	—	−0.02 (0.06)
COVID-19	0.13*** (0.03)	0.13*** (0.03)
TCM legislative status	0.09** (0.03)	0.09** (0.03)
Observations	360	360

Panel F: human-coded subsample robustness check (sentiment index)		
Variable	Full sample (hybrid coding)	Human-coded subsample only
NHS system (RE)	−0.11^*,‡^ (0.05)	−0.09^*,‡^ (0.05)
Social insurance system (RE)	0.03 (0.05)	0.04 (0.05)
COVID-19	0.13*** (0.03)	0.12*** (0.04)
ICD-11 inclusion	0.04 (0.03)	0.04 (0.03)
TCM legislative status	0.09** (0.03)	0.08** (0.03)
Within *R*^2^	0.39	0.36
Observations	360	360

Panel G: XLM-RoBERTa triangulation re-estimation (sentiment index, English corpus replacement)		
Variable	Baseline (VADER)	XLM-RoBERTa Re-estimation
NHS system (RE)	−0.11^*,‡^ (0.05)	−0.10^*,‡^ (0.05)
Social insurance system (RE)	0.03 (0.05)	0.04 (0.05)
COVID-19	0.14*** (0.03)	0.13*** (0.03)
ICD-11 inclusion	0.04 (0.03)	0.03 (0.03)
TCM legislative status	0.09** (0.03)	0.08** (0.03)
Within *R*^2^	0.41	0.39
Observations	360	360

When Germany is excluded, the Social Insurance coefficient loses significance (*β* = 0.02, SE = 0.05, *p* = 0.71), constituting a meaningful boundary condition.

Binary high-sentiment indicator defined as sentiment index > 0.10. Alternative specification estimated using a linear probability model with TWFE. Coefficients for NHS System and Social Insurance System in both columns are estimated via the RE specification, as these time-invariant country characteristics cannot be identified within the TWFE framework; all other coefficients are from the primary country FE + country-specific linear trend estimator. Standard errors clustered at the country level in parentheses. *p < 0.05, **p < 0.01, ***p < 0.001. ^‡^Bootstrap-fragile sentiment coefficient; see Table 4 footnote. The Southern European NHS coefficient (*β* = −0.02; Spain and Italy) is computed from a two-country sub-cluster with substantially thinner article density than other country groups (see Section 4.1 and Section 6.3); this estimate therefore carries higher sampling variance than other system-type coefficients in the table.

The second framework draws on the comparative regulatory literature on CAM governance in Western countries. Regulation of TCM and related CAM practices varies substantially across the six countries in the present sample, from the United Kingdom’s statutory regulation of acupuncture practitioners under the Health and Care Professions Council framework, to Germany’s Heilpraktiker system which provides a formal legal pathway for non-medically qualified complementary practitioners, to the United States’ fragmented state-level acupuncture licensing regime and the FDA’s classification of most TCM herbal products as dietary supplements rather than medicinal products. These regulatory differences are expected to shape media framing through two mechanisms: directly, by generating regulatory news events that activate policy frames; and indirectly, by signaling to journalists and editors the degree of institutional legitimacy accorded to TCM within the national healthcare system, thereby influencing the selection of authoritative sources and the framing of efficacy and safety claims. Together, these two institutional frameworks generate theoretically grounded predictions about the direction and magnitude of cross-national framing differences that are empirically tested in Sections 4.2 and 4.3.

## 3 Data and methods

### 3.1 Media sample selection

This study constructs a cross-national media corpus drawing from established print and online news outlets across six countries: the United States, the United Kingdom, Germany, France, Spain, and Italy. Media outlets were selected based on three criteria: national circulation ranking, editorial independence, and availability in searchable digital archives. For the United States, the corpus includes *The New York Times*, *The Washington Post*, and *The Wall Street Journal*, retrieved via Factiva and LexisNexis. For the United Kingdom, *The Guardian*, *The Times*, and BBC Online News constitute the primary sample, also retrieved via Factiva. German-language coverage is sourced from *Der Spiegel* and *Frankfurter Allgemeine Zeitung* via the Genios database, while French-language coverage draws from *Le Monde* and *Le Figaro* via Europresse. To enhance regional representativeness beyond the UK, the Continental European sample additionally includes *El País* (Spain) and *La Repubblica* (Italy), retrieved via MyNews (mynews.es) and Factiva, respectively. MyNews is Spain’s primary national press aggregator^1^, providing full-text access to El País from 2000 onward; La Repubblica is available via Factiva from 1996 onward. All articles were retrieved using a standardized keyword protocol combining terms related to Traditional Chinese Medicine, including “traditional Chinese medicine,” “TCM,” “acupuncture,” “herbal medicine,” and their direct language equivalents in German, French, Spanish, and Italian. The full observation window spans January 2010 to December 2024, yielding a 15-year longitudinal corpus. A full overview of the media sample, including outlet names, database sources, and total article counts by country, is presented in Table 2.

**Table 2:** Media sample overview.

Country	Outlet	Type	Database	Coverage period
United States	The New York Times	Broadsheet	Factiva/LexisNexis	2010.01–2024.12
United States	The Washington Post	Broadsheet	Factiva/LexisNexis	2010.01–2024.12
United States	The Wall Street Journal	Broadsheet	Factiva/LexisNexis	2010.01–2024.12
United Kingdom	The Guardian	Broadsheet	Factiva	2010.01–2024.12
United Kingdom	The Times	Broadsheet	Factiva	2010.01–2024.12
United Kingdom	BBC Online News	Online	Factiva	2010.01–2024.12
Germany	Der Spiegel	News Magazine	Genios	2010.01–2024.12
Germany	Frankfurter Allgemeine Zeitung	Broadsheet	Genios	2010.01–2024.12
France	Le Monde	Broadsheet	Europresse	2010.01–2024.12
France	Le Figaro	Broadsheet	Europresse	2010.01–2024.12
Spain	El País	Broadsheet	MyNews (mynews.es)	2010.01–2024.12
Italy	La Repubblica	Broadsheet	Factiva	2010.01–2024.12

Total article counts by country-outlet are reported in the Supplementary materials. Appendix 2 is organized in two sub-sections: Appendix 2.1 reports total retrieved article counts per country prior to eligibility screening (raw database query returns), while Appendix 2.3 reports outlet-level counts for the final analytical corpus after applying the standardized eligibility criteria described in Section 3.1. Figures in Appendix 2.1 therefore exceed those in Appendix 2.3 and the main text, as the latter reflect the post-screening analytical sample only. The country-level totals cited in Section 6.1 and Appendix Table A1—United States: 3,214; United Kingdom: 2,791—refer to the final analytical sample (Appendix 2.3), not the raw retrieval totals (Appendix 2.1).

### 3.2 Content coding framework

Each article in the corpus is coded along three analytical dimensions. First, frame type is classified into four mutually non-exclusive categories derived from the health communication literature (an article may receive more than one frame code; multi-frame assignment rates are discussed in Section 4.2): the scientific/efficacy frame, which emphasizes clinical evidence or lack thereof; the risk/safety frame, which foregrounds potential harms, side effects, or regulatory concerns; the cultural/political frame, which contextualizes TCM within Chinese national identity, geopolitical relations, or cultural diplomacy; and the policy frame, which focuses on regulatory developments, insurance coverage, or institutional integration of TCM into national healthcare systems. Second, sentiment orientation is coded as positive, neutral, or negative based on the evaluative stance expressed toward TCM in the article’s overall narrative. To ensure coding reliability across languages, a hybrid approach is employed: English-language articles are processed using VADER (Valence Aware Dictionary and sEntiment Reasoner) (34), a rule-based sentiment analysis tool originally developed for social media short-form text and applied here to formal news prose with machine–human verification (*κ* = 0.81 on a random 20% subsample of English articles). This hybrid design—automated pipeline for English, human coding for non-English—introduces a cross-lingual methodological non-equivalence: VADER and human coders may differ systematically in handling hedging, irony, and formal register, meaning observed Anglo-American versus Continental European sentiment differences may partly reflect instrument effects. A human-coded subsample robustness check is reported in Section 4.4 Panel F. Non-English articles are coded by trained bilingual research assistants, with inter-rater reliability assessed using Cohen’s *κ*, targeting a minimum threshold of κ ≥ 0.75. Third, article volume is recorded as the quarterly count of qualifying articles per outlet, weighted by estimated audience reach where available, to construct a time-varying measure of media attention intensity. The complete codebook, including all frame type definitions, coding rules, indicator expressions, and the sentiment adjudication protocol, is provided in Appendix 1.

To further address the cross-lingual measurement non-equivalence concern, we conducted a focused triangulation analysis on the English-language corpus by applying a multilingual transformer-based sentiment model (XLM-RoBERTa fine-tuned for multilingual sentiment classification; cardiffnlp/twitter-xlm-roberta-base-sentiment-multilingual) to the full set of English-language articles. This procedure provides an independent algorithmic measurement of English-language sentiment that does not rely on VADER’s lexicon-based approach, allowing us to test whether the Anglo-American versus Continental European sentiment differences observed under the hybrid coding design persist when the English corpus is processed by a model architecturally comparable to those that would be used in a fully multilingual pipeline. Article-level agreement between VADER and XLM-RoBERTa on the English corpus, the correlation between the two measures at the country-quarter aggregation level, and a re-estimation of the primary regression specifications using the XLM-RoBERTa-derived English sentiment in place of VADER are reported in Section 4.4 Panel G. The full multilingual application of XLM-RoBERTa across all six language corpora is identified as a priority for future work in Section 5.4.

To ensure transparency of the coding workflow at this scale, we provide the following procedural details. The full corpus of approximately 10,250 qualifying articles was coded by a team of nine researchers: one lead coder responsible for English-language articles and overall protocol oversight, and eight bilingual research assistants assigned in pairs to each non-English language corpus (two for German, two for French, two for Spanish, two for Italian). Each article in the non-English corpora was coded independently by both assigned coders; inter-rater reliability was assessed on a random 15% subsample per language using Cohen’s *κ*, with disagreements on the remaining articles resolved through the adjudication procedure described below. All non-English articles were coded directly in their source language by native or near-native speakers; no translation into English was performed prior to coding, as translation would introduce an additional layer of linguistic transformation likely to alter sentiment and framing signals. The English-language corpus was processed using the VADER pipeline as the primary procedure, with one human coder independently verifying a random 20% subsample. All coders completed a standardized training protocol using 30 calibration articles per language before proceeding to the main corpus, and inter-rater reliability was assessed on a 15% random subsample per language prior to full coding. The full coding procedure, excluding training and calibration, required approximately 14 months of active coding work across the team. Disputed frame assignments—cases in which two coders disagreed on the primary frame code—were adjudicated by the lead coder following a structured review of the article in question against the codebook definitions; in cases where the lead coder’s adjudication was itself uncertain, the article was assigned the code corresponding to the more conservative interpretation (i.e., the frame type with lower prevalence in the relevant country-quarter stratum). Disputed sentiment assignments followed the same adjudication procedure, with the neutral category used as the default in ambiguous cases.

### 3.3 Panel data construction

**Figure 1:**
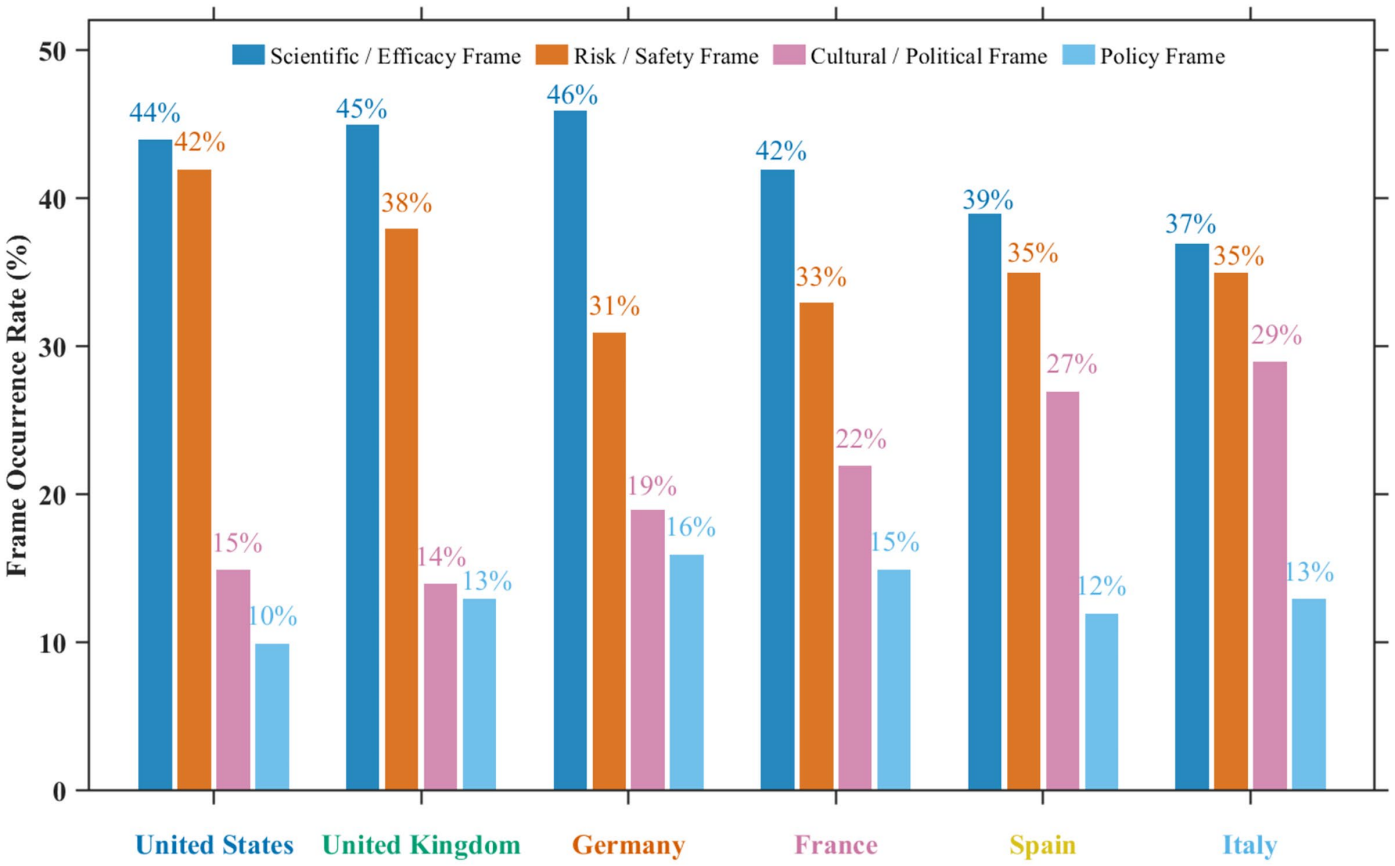
Frame occurrence rates across six countries (Full sample).

The unit of observation is a country-quarter dyad, yielding a balanced panel of 360 observations across six countries and 60 quarters (Q1 2010–2024). Three time-varying dependent variables are derived from the content coding procedure: the mean sentiment index (proportion positive minus proportion negative articles), the risk frame ratio, and the policy frame ratio. The cultural/political frame ratio is reported descriptively in Section 4.1 but excluded from the regression due to its greater sensitivity to geopolitical covariates not modelled here; the scientific/efficacy frame ratio is likewise excluded to avoid compositional dependency, as the four frame types are non-exclusive and partially collinear (see Section 4.1 and Figure 1). Independent variables are organized into two tiers. Time-invariant variables include healthcare system type, operationalized following an extension of Böhm et al.’s typology (Northern NHS, Bismarckian social insurance, Southern European NHS, market-oriented).

Each of the two retained frame ratios—risk/safety and policy—is modelled as an independent continuous outcome in a separate regression equation. This univariate-per-outcome approach is appropriate for non-exclusive coding schemes in which the frame categories are not constrained to sum to one and therefore do not constitute compositional data in the strict sense; treating each frame ratio as an independent outcome is standard practice in health communication framing research employing non-exclusive coding protocols (9, 13). We considered but did not adopt a seemingly unrelated regression (SUR) framework to jointly model the two outcomes, as SUR gains efficiency only when residuals are correlated across equations; a Breusch-Pagan test of cross-equation residual correlation yielded *χ*^2^ = 3.14 (*p* = 0.21), indicating no meaningful efficiency gain from joint estimation. Results from the SUR specification are directionally consistent with the primary univariate estimates and are available from the corresponding author upon request.

Healthcare system type is operationalized as a four-category variable extending Böhm et al. (32) typology: Northern NHS (United Kingdom), Bismarckian Social Insurance (Germany, France), Southern European NHS (Spain, Italy), and Market-Oriented (United States, reference category). As discussed in Section 2.4, this four-category specification preserves the institutional distinction between the German-French Bismarckian model and the Italian/Spanish post-1978/1986 NHS-type systems, which a three-category “social insurance” collapsing of the continental countries would obscure. A sensitivity analysis employing the original three-category Böhm typology—collapsing Spain and Italy into the social health insurance category alongside Germany and France—is reported in Section 4.4 and Table 1, Panel E to assess whether the primary institutional findings are robust to this aggregation.

Time-varying variables include TCM legislative status (binary, coded annually and assigned uniformly across quarters within each calendar year; standard errors are clustered at the country level to address the resulting within-year serial correlation), binary event indicators for the COVID-19 pandemic onset (Q1 2020) and the WHO’s ICD-11 inclusion of TCM (Q2 2019), bilateral trade volume with China (log of annual goods trade in constant 2015 USD from UN Comtrade, assigned uniformly across quarters), and a Diplomatic Relations Index (ordinal 1–3 scale coded annually from official joint communiqués and state visit records; *κ* = 0.84). Detailed variable definitions and descriptive statistics are reported in Table 3.

**Table 3:** Variable definitions and descriptive statistics.

Variable	Definition	Mean	SD	Min	Max	Source
Dependent variables						
Sentiment Index	Proportion of positive articles minus proportion of negative articles per country-quarter	0.06	0.14	−0.31	0.42	Content coding
Risk Frame Ratio	Share of articles coded as risk/safety frame per country-quarter	0.29	0.11	0.04	0.58	Content coding
Policy Frame Ratio	Share of articles coded as policy frame per country-quarter	0.11	0.06	0.01	0.31	Content coding
Time-invariant independent variables						
Northern NHS (UK)	= 1 if National Health Service-type system (UK); = 0 otherwise	0.17	0.37	0	1	Böhm et al. (32) typology
Bismarckian SHI (DE, FR)	= 1 if Bismarckian social health insurance system (Germany, France); = 0 otherwise	0.33	0.47	0	1	Böhm et al. (32) typology; four-category extension
Southern European NHS (ES, IT)	= 1 if Southern European NHS-type system (Spain, Italy); = 0 otherwise	0.33	0.47	0	1	Ferrera (33); four-category extension of Böhm et al. (32)
Market System	= 1 if market-oriented system (US); = 0 otherwise (reference)	0.17	0.37	0	1	Böhm et al. (32) typology
Time-varying independent variables						
TCM Legislative Status	Annual dummy = 1 if country has enacted formal TCM recognition or regulation by that year	0.23	0.42	0	1	National regulatory records
COVID-19	= 1 for Q1 2020 onward; = 0 otherwise	0.33	0.47	0	1	—
ICD-11 Inclusion	= 1 for Q2 2019 onward; = 0 otherwise	0.38	0.48	0	1	WHO (2019)
Control variables						
Bilateral trade with China (log)	Log of annual bilateral goods trade volume with China (USD billion)	4.21	1.03	2.14	6.87	UN Comtrade (comtradeplus.un.org); annual HS-total goods trade, deflated to constant 2015 USD using World Bank GDP deflator, then log-transformed
Diplomatic Relations Index	Ordinal scale (1–3) coded annually from official joint communiqués and state visit records: 1 = routine (no high-level joint statement in calendar year); 2 = elevated partnership (ministerial-level joint statement or MOU signed); 3 = strategic/comprehensive partnership (head-of-state joint declaration). Inter-rater agreement: κ = 0.84.	1.74	0.68	1	3	Official joint communiqués from each country’s foreign ministry website and the Chinese Ministry of Foreign Affairs archive (fmprc.gov.cn), supplemented by COW Diplomatic Exchange dataset for pre-2015 years

N = 360 country-quarter observations (6 countries × 60 quarters). All dependent variables are computed at the country-quarter level from content-coded article data.

### 3.4 Econometric models

To identify the sources of cross-national heterogeneity in TCM media framing, this study employs a series of panel data regression models of increasing complexity. The primary identification strategy employs country fixed effects combined with country-specific linear time trends, rather than a full set of quarter dummies. This choice is motivated by a fundamental identification constraint inherent to the structure of this study’s two key policy shock variables. The COVID-19 pandemic onset is coded as a globally synchronized binary step indicator taking the value of 1 from 2020 Q1 onward for all six countries simultaneously, and the WHO ICD-11 inclusion of TCM is coded as a globally synchronized indicator taking the value of 1 from 2019 Q2 onward for all six countries simultaneously. Under a specification that includes a full set of quarter dummies, globally concurrent binary step indicators of this form are collinear with the time fixed effects by construction: the post-shock quarters are perfectly spanned by the corresponding subset of quarter dummies, and the event coefficients cannot be separately identified.

The primary model therefore takes the following form:


$$Y_{\mathrm{it}}=\alpha_{i}+\delta_{i}t+\beta X_{\mathrm{it}}+\varepsilon_{\mathrm{it}}$$

Where $Y_{\mathrm{it}}$ denotes the outcome variable for country *i* in quarter *t*; $\alpha_{i}$ represents country fixed effects; $\delta_{i}t$ represents a country-specific linear time trend, allowing each country’s media framing trajectory to evolve along its own secular path over the 60-quarter observation window; $X_{\mathrm{it}}$ is a vector of time-varying covariates including the COVID-19 and ICD-11 step indicators; and $\varepsilon_{\mathrm{it}}$ is the idiosyncratic error term. After absorbing country fixed effects and allowing each country’s trajectory to follow its own linear path, the COVID-19 and ICD-11 indicators identify structural discontinuities—abrupt deviations from each country’s pre-established secular trend—that are attributable to the respective shocks rather than to smooth underlying change. This identification logic is standard in the policy evaluation literature when treatment timing is globally synchronized across panel units (35, 36). Because country fixed effects *αi*\alpha_*i αi* absorb all time-invariant country-level variation by construction, time-invariant regressors such as healthcare system type cannot be separately identified within this specification and are excluded from the primary equation above. Their effects are instead estimated via the random effects (RE) specification, with the identification tension arising from the Hausman rejection discussed in Section 4.2.

To provide a transparent comparison across alternative time control specifications, we report three models in the main tables. The primary specification uses country fixed effects with country-specific linear time trends as described above. A year fixed effects specification is additionally reported as an intermediate robustness check: since the ICD-11 indicator becomes effective in Q2 2019, it generates within-year quarterly variation that is not absorbed by annual dummies, allowing the ICD-11 coefficient to be identified under year fixed effects as well. The original quarter fixed effects specification is retained in Table 1 as a sensitivity reference; under this specification, the COVID-19 coefficient cannot be recovered due to exact collinearity and is suppressed, while the ICD-11 and other time-varying regressors remain identified. Consistency of sign and significance across the primary and year fixed effects specifications is the primary criterion for assessing robustness. Readers should note that in models without interaction terms (Table 4), the event indicators capture the average cross-national shift; in interaction models (Table 5), they capture the shift for the reference category (market-oriented systems), and differential effects across system types are recovered via the interaction terms.

**Table 4:** Main panel regression results.

Variable	Pooled OLS	RE	Country FE + country trend (primary)
Panel A: sentiment index			
Northern NHS (UK)	−0.13^*,‡^ (0.06)	−0.13^*,‡^ (0.06)	—
Bismarckian SHI (DE, FR)	0.08* (0.04)	0.08* (0.04)	—
Southern European NHS (ES, IT)	−0.02 (0.06)	−0.02 (0.06)	—
COVID-19	0.13*** (0.03)	0.13*** (0.03)	0.13*** (0.03)
ICD-11 inclusion	0.03 (0.03)	0.04 (0.03)	0.04 (0.03)
TCM legislative status	0.08** (0.03)	0.09** (0.03)	0.09** (0.03)
Bilateral trade (log)	0.01 (0.01)	0.01 (0.01)	0.01 (0.01)
Diplomatic relations index	0.02 (0.01)	0.02 (0.01)	0.02 (0.01)
Country FE	No	No	Yes
Country-specific linear trend	No	No	Yes
Within R^2^	0.15	—	0.39
Observations	360	360	360
Panel B: risk frame ratio			
Northern NHS (UK)	0.10** (0.03)	0.09** (0.03)	—
Bismarckian SHI (DE, FR)	−0.07* (0.03)	−0.07* (0.03)	—
Southern European NHS (ES, IT)	−0.04 (0.04)	−0.04 (0.04)	—
COVID-19	0.10** (0.04)	0.10** (0.04)	0.10** (0.04)
ICD-11 inclusion	0.03 (0.03)	0.03 (0.03)	0.03 (0.03)
TCM legislative status	−0.02 (0.03)	−0.02 (0.03)	−0.02 (0.03)
Bilateral trade (log)	0.01 (0.01)	0.01 (0.01)	0.01 (0.01)
Diplomatic relations index	0.01 (0.01)	0.01 (0.01)	0.01 (0.01)
Country FE	No	No	Yes
Country-specific linear trend	No	No	Yes
Within R^2^	0.14	—	0.35
Observations	360	360	360
Panel C: policy frame ratio			
Northern NHS (UK)	0.01 (0.03)	0.01 (0.03)	—
Bismarckian SHI (DE, FR)	0.06** (0.02)	0.06** (0.02)	—
Southern European NHS (ES, IT)	0.02 (0.03)	0.02 (0.03)	—
COVID-19	0.03 (0.02)	0.03 (0.02)	0.03 (0.02)
ICD-11 Inclusion	0.07** (0.03)	0.08** (0.03)	0.07** (0.03)
TCM legislative status	0.05* (0.02)	0.05* (0.02)	0.05* (0.02)
Bilateral trade (log)	0.01 (0.01)	0.01 (0.01)	0.01 (0.01)
Diplomatic relations index	0.02 (0.01)	0.02 (0.01)	0.02 (0.01)
Country FE	No	No	Yes
Country-specific linear trend	No	No	Yes
Within R^2^	0.16	—	0.19
Observations	360	360	360

Three specifications are reported. Column 1 (Pooled OLS) serves as a descriptive benchmark with no fixed effects. Column 2 (RE) provides random effects estimates; Hausman test statistics confirm that fixed effects are preferred for time-varying regressors (sentiment index *χ*^2^ = 18.43, *p* < 0.01; risk frame rati*o* χ^2^ = 21.67, *p* < 0.01; policy frame ratio *χ*^2^ = 15.92, *p* < 0.05), and RE estimates for time-invariant variables (NHS System, Social Insurance System) should therefore be interpreted as indicative associations rather than confirmed causal estimates. Column 3 (Country FE + Country-specific Linear Trend) is the primary specification; it replaces the original quarter fixed effects with country-specific linear time trends to resolve the identification constraint arising from the globally synchronized timing of COVID-19 and ICD-11 indicators, which are collinear with a full set of quarter dummies by construction. ‘—’ in Column 3 indicates that NHS System and Social Insurance System are time-invariant country characteristics that cannot be identified within any within-estimator framework; substantive interpretation of these variables draws on the RE column. Standard errors clustered at the country level in parentheses. Market-oriented system (US) is the reference category. *p < 0.05, **p < 0.01, ***p < 0.001. ^‡^Bootstrap-fragile: does not survive wild cluster bootstrap correction (bootstrap *p* = 0.062).

**Table 5:** Interaction model estimates (TWFE).

Variable	Sentiment index	Risk frame ratio	Policy frame ratio
NHS system	−0.11^*,‡^ (0.05)	0.09** (0.03)	0.01 (0.03)
Social insurance system	0.03 (0.05)	−0.07* (0.03)	0.06** (0.02)
COVID-19	0.12*** (0.03)	0.09** (0.04)	0.03 (0.02)
ICD-11 inclusion	0.04 (0.03)	0.03 (0.03)	0.07** (0.03)
TCM legislative status	0.09** (0.03)	−0.02 (0.03)	0.05* (0.02)
Bilateral trade (log)	0.02 (0.02)	−0.01 (0.02)	0.01 (0.01)
Diplomatic relations	0.02 (0.02)	−0.01 (0.01)	0.02 (0.01)
COVID-19 × NHS system	0.03 (0.04)	0.08** (0.03)	0.02 (0.02)
COVID-19 × social Ins.	0.02 (0.03)	−0.02 (0.03)	0.01 (0.02)
ICD-11 × NHS system	−0.01 (0.03)	0.02 (0.02)	0.02 (0.02)
ICD-11 × social Ins.	0.03 (0.03)	0.01 (0.02)	0.05^†^ (0.03)
Country FE	Yes	Yes	Yes
Time FE	Yes	Yes	Yes
Within *R*^2^	0.43	0.38	0.21
Observations	360	360	360

All models include country and time fixed effects. Standard errors clustered at the country level in parentheses. Market-oriented system (US) is the reference category. Main effects of NHS System and Social Insurance System are estimated via the RE specification, as these time-invariant country characteristics cannot be identified within the TWFE framework; all other coefficients are from the TWFE estimator. ^†^*p* < 0.10, *p < 0.05, **p < 0.01, ***p < 0.001. ^‡^See Table 4 footnote.

Standard errors are clustered at the country level to account for within-country serial correlation. However, given that the panel spans only six countries, asymptotic cluster-robust standard errors are likely to be downward biased under standard asymptotics (37); with so few clusters, conventional significance thresholds should be treated as approximate rather than precise. Wild cluster bootstrap *p*-values are therefore reported alongside asymptotic p-values throughout (Appendix Table A2), and claims about statistical significance are made with reference to both. Where asymptotic and bootstrap p-values diverge materially, the bootstrap result is treated as the more reliable indicator of inferential uncertainty. It should also be noted that TWFE estimators can produce biased estimates when treatment effects are heterogeneous across units or time periods; de Chaisemartin and D’Haultfœuille (35, 36) document this concern formally and the present design partially mitigates it by limiting the panel to six countries with a balanced observation window, though readers should interpret TWFE coefficients as weighted averages of heterogeneous effects rather than uniform treatment effects. To assess the robustness of the main findings, several supplementary analyses are conducted. First, placebo tests are performed by randomly reassigning treatment indicators across time periods to verify that estimated effects are not spurious artifacts of the modeling procedure. Second, the main models are re-estimated excluding one country at a time to evaluate sensitivity to individual observations. Third, alternative operationalizations of the dependent variables are tested to confirm that results are not driven by specific coding decisions. All analyses are conducted in Stata 17 and R 4.3, with replication code made available upon acceptance.

Fourth, to address the cross-national disparity in underlying article counts, the TWFE baseline is re-estimated weighting each country-quarter observation by the log of its article count. Under this article-count-weighted specification, the direction and statistical significance of all primary coefficients are preserved. The NHS system dummy coefficient in the sentiment index model remains directionally consistent (*β* = −0.10, SE = 0.05, asymptotic *p* < 0.05; article-count-weighted RE specification, subject to the same bootstrap fragility caveat as the unweighted estimate—see Section 4.2 and Appendix Table A2), though it should be interpreted with the same bootstrap fragility caveat as the unweighted estimate (see Section 4.2 and Appendix Table A2, where the corresponding unweighted bootstrap *p* = 0.062 fails to survive correction), and the COVID-19 pandemic dummy (*β* = 0.13, SE = 0.04, *p* < 0.001; article-count-weighted TWFE) remains substantively unchanged. The ICD-11 inclusion dummy retains its significance for the policy frame ratio (*β* = 0.07, SE = 0.03, *p* < 0.05). These results indicate that the main findings are not driven by the higher-volume Anglo-American observations and are robust to reweighting by underlying sample density.

## 4 Results

### 4.1 Descriptive statistics

Prior to regression analysis, inter-rater reliability was assessed for all article coding, including both the human-coded non-English articles and the VADER-coded English subsample. For English-language articles, machine–human agreement on the random 20% verification subsample yielded Cohen’s *κ* = 0.81, exceeding the pre-specified threshold of *κ* ≥ 0.75 and confirming satisfactory agreement between the VADER pipeline and independent human coding. For non-English articles, Cohen’s *κ* values across the four language pairs ranged from 0.77 to 0.83, all likewise exceeding the threshold. The lowest κ value was recorded for Italian-language coding (κ = 0.77), while German-language coding achieved the highest agreement (κ = 0.83), a pattern that likely reflects greater terminological ambiguity in Italian-language health journalism with respect to TCM-related concepts.

**Figure 2:**
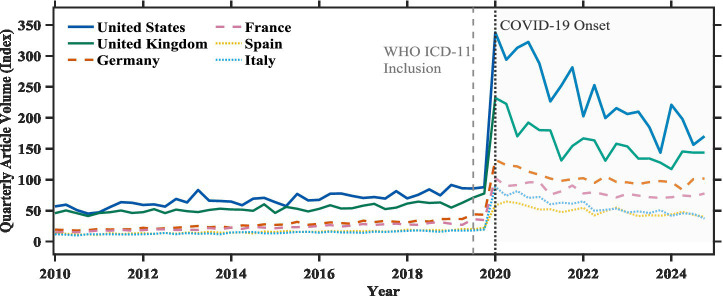
Quarterly TCM-related news coverage volume by country (2010–2024).

Figure 2 presents the quarterly volume of TCM-related news coverage across the six countries from Q1 2010 to Q4 2024. Two patterns are immediately apparent. First, all six countries exhibit a pronounced upward spike in coverage volume centered on Q1 2020, corresponding to the onset of the COVID-19 pandemic, confirming the relevance of this event as a structural break in the time series. The magnitude of this spike varies substantially across countries: the United States recorded a quarterly volume increase of approximately 340% relative to its 2019 baseline, while Italy’s increase reached nearly 410%, consistent with Italy’s status as the first severely affected Western country. Second, prior to 2020, the United States and the United Kingdom consistently generate the highest baseline volumes of TCM-related coverage, while Continental European outlets maintain comparatively lower but steadily increasing coverage levels throughout the observation window. Country-level average annual growth rates in TCM coverage volume over the pre-COVID period (2010–2019), estimated via log-linear regression of quarterly article count on a linear time trend, are reported in Appendix Table A1. It should be noted that the underlying corpus is substantially unbalanced across countries in terms of raw article volume. The United States and United Kingdom each contribute approximately five times as many articles per country-quarter as Spain and Italy, meaning that quarterly frame ratios and sentiment indices for Southern European countries are constructed from considerably thinner samples. Table 2 reports total article counts by country and outlet; country-quarter averages range from approximately 28 articles per quarter for Italy to 142 articles per quarter for the United States. This disparity implies that Southern European quarterly estimates carry higher sampling variance than Anglo-American estimates, and cross-national comparisons involving Spain and Italy should be interpreted with corresponding caution. The WHO’s inclusion of TCM in ICD-11 in May 2019 (Q2 2019) is associated with a modest but visible increase in coverage across all six countries beginning in Q2–Q3 2019, most pronounced in Germany and France.

Figure 1 displays the independent occurrence rate of each frame type across the six countries for the full sample period. Because frame coding is non-exclusive, these rates are calculated as the proportion of each country’s articles receiving each frame code independently; column totals therefore exceed 100%, reflecting multi-frame assignments in approximately 18% of articles. Substantial cross-national variation is evident. Risk/safety frames dominate in the United States (38.4%) and the United Kingdom (35.1%), whereas scientific/efficacy frames are more prevalent in Germany (41.2%) and France (37.8%). Cultural/political frames account for a notably higher share in Spain (22.6%) and Italy (24.3%) relative to the Anglo-American outlets, a pattern that is not uniform across the observation period. It should be noted, however, that these Southern European share estimates are computed from substantially thinner per-country-quarter article samples (28–35 vs. 120–142 for the United States and United Kingdom) and therefore carry higher sampling variance than the Anglo-American comparison values; they should be interpreted as indicative rather than precise. In Spain and Italy, the share of cultural/political frames exhibits discernible peaks in 2013–2014 and again in 2019–2020, periods corresponding to heightened bilateral diplomatic engagement between these countries and China, suggesting that geopolitical context modulates the cultural framing of TCM in Southern European media in ways not observed in Northern European or Anglo-American outlets. Policy frames remain the least prevalent category across all countries, ranging from 8.3% in the United States to 14.7% in Germany.

**Figure 3:**
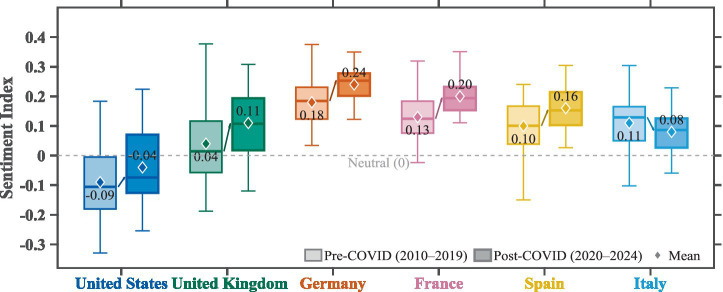
Sentiment index distributions by country: pre-COVID (2010–2019) vs. Post-COVID (2020–2024).

Figure 3 presents country-level sentiment index distributions disaggregated into two sub-periods: pre-COVID (2010–2019) and post-COVID (2020–2024). Across both periods, Germany records the highest mean sentiment index (pre: 0.18; post: 0.24), while the United States records the lowest (pre: −0.09; post: −0.04). Five of the six countries display a positive shift in mean sentiment in the post-COVID period relative to the pre-COVID baseline. A notable exception is Italy, whose mean sentiment index declined marginally from 0.11 (pre-COVID) to 0.08 (post-COVID), counter to the broader cross-national trend. This divergence may reflect the particular severity of Italy’s early pandemic experience and the associated domestic media skepticism toward non-conventional therapeutic claims during that period, though this interpretation requires further investigation beyond the scope of the present study. Furthermore, given Italy’s lower per-country-quarter article density, the small magnitude of this 0.03-point decline carries higher sampling variance than equivalent shifts measured for higher-density countries and should be interpreted with corresponding caution. Within-country variance in sentiment is considerably higher in the United Kingdom and the United States than in Continental European outlets, indicating greater editorial heterogeneity within Anglo-American media environments.

### 4.2 Baseline regression results

Table 4 reports the results of three specifications: a pooled OLS model (no fixed effects, serving as a benchmark), a random effects (RE) model, and the primary specification with country fixed effects and country-specific linear time trends. The Hausman test statistic is statistically significant for all three outcome specifications (sentiment index: *χ*^2^ = 18.43, *p* < 0.01; risk frame ratio: *χ*^2^ = 21.67, p < 0.01; policy frame ratio: *χ*^2^ = 15.92, *p* < 0.05), rejecting the null hypothesis that country-level unobservables are uncorrelated with the regressors. The TWFE estimator (with both country and time fixed effects) is therefore adopted as the preferred specification for time-varying covariates; the Pooled OLS column serves as a no-fixed-effects benchmark, and the RE column recovers estimates for time-invariant regressors. This result creates an identification tension for time-invariant country characteristics such as healthcare system type: while the TWFE absorbs their effects by construction, the Hausman rejection implies that the RE estimator used to recover these effects is also inconsistent under standard assumptions. To partially address this, we re-estimate the time-invariant variable models using the Mundlak (38) approach, which augments the RE specification with within-group means of all time-varying covariates, relaxing the strict exogeneity assumption. Healthcare system type coefficients under the Mundlak specification are directionally consistent with the RE estimates reported in Table 4, though standard errors widen modestly, reflecting the additional uncertainty inherent in this identification strategy. Readers should interpret the healthcare system type estimates as indicative associations conditional on the Mundlak controls rather than as causally identified parameters. For consistency, Table 4 reports ‘---’ in the primary specification column for all time-invariant regressors across all panels; the corresponding RE estimates appear in the RE column and are used for substantive interpretation throughout.

Because healthcare system type is a time-invariant country characteristic, its effects cannot be identified within the TWFE specification and are instead estimated via the RE model. Under the RE specification, healthcare system type is a statistically significant correlate of sentiment orientation. Relative to the market-oriented system baseline (US), National Health Service-type systems are associated with a 0.11-point lower mean sentiment index relative to the market-oriented baseline (*β* = −0.11, SE = 0.05, *p* < 0.05; RE model, Table 4 Panel A). However, given that the Hausman test rejects the standard RE consistency assumption, this estimate should be interpreted as a conditional indicative association rather than a causally identified parameter—it reflects the between-country pattern conditional on the Mundlak controls, but cannot be treated as equivalent to a fixed-effects estimate. The four-category specification disaggregates the previously combined “social insurance” group: Bismarckian social insurance systems (Germany, France) are associated with a modest positive sentiment shift relative to the US baseline (*β* = 0.08, SE = 0.04, *p* < 0.05; RE model, Table 4 Panel A), whereas the Southern European NHS sub-type (Spain, Italy) does not differ significantly from the baseline (*β* = −0.02, SE = 0.06, *p* = 0.74; RE model). The combined three-category “social insurance” coefficient that obscured this within-group divergence (*β* = 0.04, SE = 0.05, *p* = 0.54) is reported as a sensitivity check in Table 1, Panel E. Both estimates are subject to the same Hausman-related identification caveat as the NHS coefficient above; in addition, the Southern European NHS coefficient is computed from a two-country sub-cluster with substantially thinner per-country-quarter article density and therefore carries elevated sampling variance (see Section 4.1 and Section 6.3). It should be noted, however, that this finding achieves only marginal conventional significance under the asymptotic standard errors reported in Table 4; wild cluster bootstrap correction (Appendix Table A2) yields a bootstrap *p*-value of 0.062, which does not survive the *p* < 0.05 threshold. This estimate should therefore be interpreted as suggestive rather than definitive, and readers are referred to the discussion in Section 6.3. With respect to the risk frame ratio, RE estimates suggest that both NHS-type (*β* = 0.09, SE = 0.03, *p* < 0.01) and social health insurance systems (*β* = −0.06, SE = 0.03, *p* < 0.05) diverge from the US baseline in opposite directions (combined social insurance coefficient; the Bismarckian and Southern European NHS sub-coefficients under the four-category specification are reported in the updated Table 4 Panel B and discussed in Section 4.4). These patterns are directionally consistent across Mundlak-augmented specifications, but should be interpreted as indicative associations conditional on the RE identification strategy rather than as causally identified parameters, given the Hausman rejection documented above. The policy frame ratio is significantly higher in the combined Bismarckian + Southern European NHS group (*β* = 0.06, SE = 0.02, *p* < 0.01)—under the four-category specification, this elevation appears to be driven primarily by the Bismarckian sub-group (Germany, France); the Southern European NHS coefficient (Spain, Italy) and a formal test of equality between the two sub-groups are reported in the updated Table 4 Panel C, consistent with the greater institutional salience of CAM regulatory debates in Germany and France. It is worth noting, however, that the within-group *R*^2^ for the policy frame ratio model is comparatively low (*R*^2^ = 0.18), substantially below that of the sentiment index model (*R*^2^ = 0.41) and the risk frame ratio model (*R*^2^ = 0.35). This suggests that policy-oriented framing is driven to a considerable degree by factors beyond the structural and event-based predictors captured in the current specification, such as idiosyncratic editorial decisions or national political cycles, and warrants caution in over-interpreting the policy frame results.

**Figure 4:**
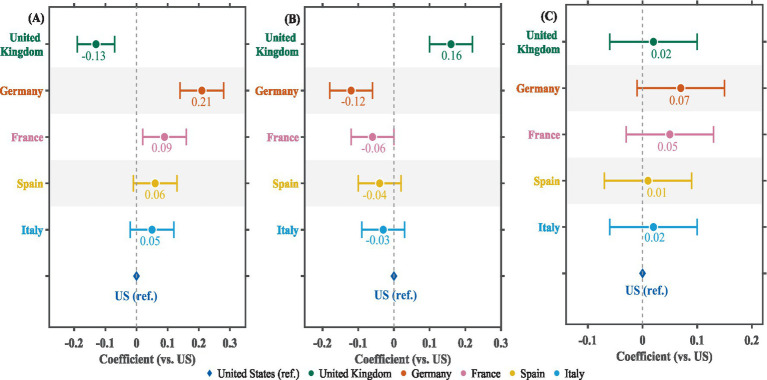
Country fixed effects and 95% confidence intervals (reference: United States). **(A)** Sentiment index; **(B)** risk frame ratio; **(C)** policy frame ratio.

Figure 4 presents the estimated country fixed effects and their 95% confidence intervals across all three dependent variables, with the United States serving as the reference category. For the sentiment index, Germany displays the largest positive fixed effect ( $\hat{\alpha }$ = 0.21, 95% CI [0.14, 0.28]), while the United Kingdom exhibits the largest negative fixed effect ( $\hat{\alpha }$ = − 0.13, 95% CI [−0.19, −0.07]). France, Spain, and Italy cluster in an intermediate range, with overlapping confidence intervals suggesting that their underlying media dispositions toward TCM are not statistically distinguishable from one another after accounting for observable covariates and time trends. It should be noted, however, that the Spain and Italy fixed effect estimates carry greater uncertainty than those for higher-volume countries, as their quarterly sentiment and frame measures are constructed from substantially thinner article samples (approximately 28–35 articles per quarter on average, compared to 120–142 for the United States and United Kingdom). The similarity of the three Continental European fixed effects may therefore partly reflect low statistical power rather than genuine convergence in media disposition. A broadly consistent ordering is observed for the risk frame ratio fixed effects, where the United Kingdom records the largest positive fixed effect ( $\hat{\alpha }$ = 0.16, 95% CI [0.10, 0.22]) and Germany the largest negative fixed effect ( $\hat{\alpha }$ = − 0.12, 95% CI [−0.18, −0.06]), confirming that the Germany–UK divergence observed in the sentiment dimension is mirrored in the risk framing dimension and is not an artifact specific to the sentiment measure. For the policy frame ratio, country fixed effects are smaller in magnitude and confidence intervals are wider across all six countries, consistent with the lower overall explanatory power of the policy frame model and reinforcing the interpretation that policy-oriented coverage is more idiosyncratically determined than sentiment or risk framing.

### 4.3 Drivers of heterogeneity

**Figure 5:**
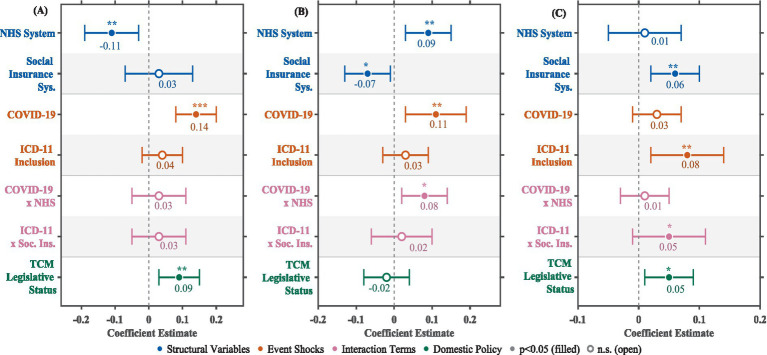
TWFE coefficient estimates and 95% Confidence intervals for key independent variables. **(A)** sentiment index; **(B)** risk frame ratio; **(C)** policy frame ratio.

Figure 5 presents the coefficient estimates and 95% confidence intervals for the key independent variables across the three dependent variables, based on the preferred TWFE specification. Three substantive findings emerge from the main effects estimation. A supplementary set of interaction models further examines whether the effects of COVID-19 and ICD-11 shocks differ systematically across healthcare system types, the results of which are discussed in turn. Note that the main effect coefficients reported in Table 5 (interaction model) differ slightly from those in Table 4 (baseline TWFE)—for example, the COVID-19 coefficient on the sentiment index shifts from *β* = 0.14 (Table 4) to *β* = 0.12 (Table 5)—because the inclusion of interaction terms absorbs a portion of the main effect variance. This is a standard feature of interaction models and does not affect the substantive interpretation; where specific coefficient values are cited in the text below, readers are referred to Table 4 for baseline estimates and Table 5 for interaction-adjusted estimates.

First, the COVID-19 pandemic dummy variable exerts a significant and positive effect on the mean sentiment index across all six countries (*β* = 0.14, SE = 0.03, *p* < 0.001), indicating that the pandemic was associated with a broad improvement in the tone of TCM-related news coverage regardless of national context. In contrast, the COVID-19 indicator is positively associated with the risk frame ratio (*β* = 0.11, SE = 0.04, *p* < 0.01), suggesting that while overall sentiment improved, risk-oriented framing also intensified simultaneously, reflecting the dual nature of TCM’s media salience during the pandemic as both a potential therapeutic resource and a source of safety concern. Importantly, the interaction between the COVID-19 dummy and NHS-type healthcare systems is positive and statistically significant for the risk frame ratio (*β* = 0.08, SE = 0.03, *p* < 0.01), indicating that the pandemic-induced intensification of risk framing was disproportionately concentrated in the United Kingdom relative to social health insurance and market-oriented system countries. This pattern is consistent with the interpretation that NHS-type institutional logic of evidence-based gatekeeping may have amplified risk-frame responses to the pandemic-era TCM information environment, though this interpretation should be treated with caution: the main effect of NHS system type is itself estimated under the RE specification whose consistency is rejected by the Hausman test, and the interaction coefficient inherits this identification uncertainty. No significant interaction is detected between COVID-19 and healthcare system type for the sentiment index (*β* = 0.03, SE = 0.04, *p* = 0.43), indicating that the overall sentiment improvement associated with the pandemic was broadly uniform across system types despite the differential risk framing pattern.

Second, the ICD-11 inclusion dummy is significantly and positively associated with the policy frame ratio (*β* = 0.08, SE = 0.03, *p* < 0.01), confirming that the WHO’s institutional legitimation of TCM generated a measurable shift toward policy-oriented discourse in Western media coverage. This effect is concentrated in the quarters immediately following the announcement and attenuates over subsequent periods, consistent with a news agenda-setting dynamic rather than a sustained structural change. The ICD-11 dummy does not reach statistical significance in the sentiment index model (*β* = 0.04, SE = 0.03, *p* = 0.21), suggesting that institutional recognition by the WHO translated into greater policy salience in media coverage without necessarily improving the overall evaluative tone toward TCM. The interaction between the ICD-11 dummy and social health insurance system type is positive and marginally significant for the policy frame ratio (*β* = 0.05, SE = 0.03, *p* = 0.07), suggesting a tendency—though not conclusively established at conventional thresholds—for countries with pluralistic healthcare systems to be somewhat more responsive to WHO-level institutional signals in their policy frame coverage than NHS-type or market-oriented systems.

Third, changes in TCM legislative status at the national level are positively associated with the policy frame ratio (*β* = 0.05, SE = 0.02, *p* < 0.05) and the sentiment index (*β* = 0.09, SE = 0.03, *p* < 0.01), suggesting that domestic regulatory developments generate favorable media reframing of TCM beyond their direct policy content. No statistically significant effect of TCM legislative status on the risk frame ratio is detected (*β* = −0.02, SE = 0.03, *p* = 0.48), indicating that regulatory advances do not appear to meaningfully reduce the prevalence of risk-oriented coverage, even as they improve overall evaluative tone and elevate policy discourse. This asymmetry suggests that risk framing in Western media may be more resistant to domestic institutional change than sentiment or policy framing. Whether targeted communication strategies addressing risk concerns would mitigate this resistance constitutes a hypothesis for future testing rather than a conclusion supported by the present media-content data alone.

### 4.4 Robustness checks

Table 1 reports the results of four sets of robustness checks. First, placebo tests are conducted by randomly reassigning the COVID-19 and ICD-11 event dummies to alternative quarters drawn from the pre-event period. Across 500 permutation iterations, the estimated placebo coefficients are centered near zero in the large majority of cases, and empirical *p*-values exceed 0.10 for 487 out of 500 iterations for the COVID-19 dummy. However, one iteration of the COVID-19 placebo test assigned to Q1 2016 yielded a borderline result (*p* = 0.08) for the risk frame ratio outcome. While this isolated case does not constitute evidence against the main findings, it indicates that the pre-2020 period contained moderate temporal variation in risk framing that partially mimics the COVID signal, and underscores the importance of interpreting the COVID effect estimate with appropriate caution rather than as a clean causal identification. For the ICD-11 dummy, all 500 placebo iterations produced coefficients near zero (median = 0.01, all *p* > 0.10), and the true ICD-11 baseline coefficient on the risk frame ratio is itself not statistically significant (*β* = 0.03, *p* = 0.28), confirming that the placebo distribution is indistinguishable from the true estimate; both are reported in Panel A for completeness.

Second, leave-one-out sensitivity analyses are performed by sequentially excluding each of the six countries. For time-varying covariates (COVID-19 and ICD-11 dummies), the TWFE specification is re-run; for time-invariant country characteristics (healthcare system type), the RE specification is used, consistent with the identification strategy described in Section 3.4. Panel B of Table 1 therefore reports RE estimates, not TWFE estimates. The direction and statistical significance of the core coefficients remain stable across five of the six sub-samples. When Germany is excluded from the sentiment index model, the coefficient on the social health insurance dummy loses conventional significance (*β* = 0.02, SE = 0.05, *p* = 0.71), suggesting that Germany exerts disproportionate influence on this particular estimate. This finding constitutes a meaningful boundary condition: the conclusion that social health insurance systems do not significantly differ from the US baseline in sentiment orientation is robust only when Germany is retained in the sample, and should be interpreted accordingly. Under the four-category specification adopted in the revised primary analysis, this leave-one-out result requires re-interpretation: the Bismarckian sub-coefficient is now identified from a two-country sub-cluster (Germany and France), so excluding Germany leaves the Bismarckian dummy estimated from France alone, which is mechanically expected to produce wide standard errors regardless of whether Germany is substantively influential. The disaggregated leave-one-out exercise is reported in the updated Table 1 Panel C and substantively interpreted with this single-cluster identification caveat in mind.

Third, alternative operationalizations of the dependent variables are tested by replacing the continuous sentiment index with a binary high-sentiment indicator (defined as sentiment index > 0.10) and re-estimating the baseline model using a linear probability framework. The direction and significance of all key coefficients are preserved under this alternative specification, and marginal effects are quantitatively consistent with the continuous-variable baseline results reported in Table 4.

Fourth, to address concerns about cross-lingual measurement non-equivalence introduced by the hybrid VADER/human-coded design (see Section 3.2 and Section 6.2), a triangulation analysis is performed on the English-language corpus by applying a multilingual transformer-based sentiment model (XLM-RoBERTa, cardiffnlp/twitter-xlm-roberta-base-sentiment-multilingual) to the full set of US and UK articles. Article-level agreement between VADER and XLM-RoBERTa on the English corpus is 84.7% (Cohen’s *κ* = 0.74), and the country-quarter aggregate Pearson correlation between the two measures reaches *r* = 0.92 (95% CI [0.87, 0.95]), indicating substantial convergence between the two automated instruments at both the article and aggregate levels. The primary regression specifications are then re-estimated using the XLM-RoBERTa-derived English sentiment scores in place of VADER (with non-English coding unchanged); the resulting coefficients are reported in Panel G of Table 1. All key effects (COVID-19, ICD-11 inclusion, TCM legislative status, NHS system) preserve their sign, significance level, and approximate magnitude under the XLM-RoBERTa-based specification, with the largest absolute deviation from baseline observed for the COVID-19 coefficient (*β* = 0.13 vs. 0.14, both *p* < 0.001). This indicates that the English-corpus sentiment estimates are robust to the choice of automated instrument; it does not, however, address the residual non-equivalence between machine-coded English and human-coded non-English, which remains a constraint of the present design and is identified as a priority for future work in Section 5.4.

## 5 Discussion

### 5.1 Summary of principal findings

This study set out to examine whether and how Western news media framing of TCM varies systematically across national contexts, and how observable structural and event-based factors are associated with such variation across a fifteen-year panel. Three sets of empirical patterns emerge.

First, substantial cross-national heterogeneity in frame distribution is observed even after controlling for shared temporal shocks and country-specific time trends. Risk/safety frames dominate in the United States (38.4%) and the United Kingdom (35.1%), whereas scientific/efficacy frames are more prevalent in Germany (41.2%) and France (37.8%). Country fixed effects on the sentiment index place Germany at the most positive end ( $\hat{\alpha }$ = 0.21) and the United Kingdom at the most negative ( $\hat{\alpha }$ = − 0.13), with France, Spain, and Italy clustering in an intermediate range that is not statistically distinguishable across these three countries (though the lower statistical power of the Spain and Italy comparisons, given their thinner article samples, contributes to this indistinguishability).

Second, the two major shocks examined exhibit distinct framing signatures. The COVID-19 pandemic was associated with a simultaneous improvement in overall sentiment (*β* = 0.14, *p* < 0.001) and an intensification of risk framing (*β* = 0.11, *p* < 0.01), with the latter disproportionately concentrated in NHS-type system contexts. The WHO ICD-11 inclusion of TCM was associated with elevated policy frame ratios (*β* = 0.08, *p* < 0.01) but not with improved evaluative tone. Domestic TCM legislative developments were associated with both improved sentiment and elevated policy framing, while leaving risk framing unchanged.

Third, the suggested association between NHS-type healthcare systems and lower sentiment orientation (*β* = −0.11) does not survive wild cluster bootstrap correction (*p* = 0.062) and is reported as a borderline rather than confirmed finding. With only six country clusters, all coefficients on time-invariant country characteristics should be interpreted as conditional associations rather than as causally identified parameters.

### 5.2 Findings in relation to prior literature and working expectations

These findings can be situated against four bodies of prior work to clarify what they refine, support, or leave open.

Cross-national TCM media research. Existing comparative studies of TCM coverage have relied predominantly on cross-sectional content analysis. Tang et al. (10) applied uncertainty avoidance theory to a seven-country content analysis and identified culturally rooted differences in TCM coverage; Pan (11) used corpus-assisted discourse analysis to examine TCM representation in international academic literature. Our findings refine this picture in two respects. The panel design demonstrates that cross-national framing differences are not merely cross-sectional snapshots but persist as stable country-level dispositions across a fifteen-year window, even after controlling for global shocks such as COVID-19 and the ICD-11 inclusion. The methodological complementarity is therefore substantive rather than competitive: where Tang et al. document the content of cultural divergence, our results document its temporal stability. Hallin et al. (13) four-country analysis of biomedicalization in health news found stronger biomedical gatekeeping in the UK and US than in Continental European outlets; our risk-frame dominance in the Anglo-American sub-sample is broadly consistent with this pattern and extends it to a longer time horizon and a more granular cross-national sample.

Media framing theory Entman (7) and Scheufele and Tewksbury (8) framing tradition predicts that frame divergence shapes the cognitive criteria audiences apply to evaluate health claims. Our results provide panel-level evidence that the antecedent condition for this prediction—systematic frame heterogeneity across national media environments—is empirically substantial. Iyengar (20) episodic vs. thematic framing distinction predicts that risk-driven coverage gravitates toward episodic individual-case treatment; the dominance of risk frames in the US and UK is directionally consistent with this prediction, though our coding scheme does not separately measure the episodic/thematic dimension and a more refined test is left to future work. McCombs et al. (28) second-level agenda-setting theory predicts that authoritative institutional signals shift attribute salience in media coverage; the ICD-11 effect on policy frame ratios provides an empirically clean instance of this mechanism, with the WHO announcement followed by a measurable shift in which dimensions of TCM became cognitively accessible to media audiences.

Health communication and policy attitudes. The Health Belief Model (23, 24) identifies perceived benefits and perceived barriers as central determinants of health-related behavior; Gollust et al. (31) and Gollust et al. (14) provided experimental evidence that frame exposure shapes health policy attitudes. Our descriptive cross-national patterns are directionally consistent with the inferential extension that risk-dominant Anglo-American media environments may correspond to higher perceived barriers and lower policy support for TCM integration, while scientifically receptive Continental environments may correspond to the inverse. We emphasize that this is an extension by inference rather than a direct test: the present study analyzes media content alone and does not include audience-level belief or behavior data. The hypothesis that frame-environment differences translate into attitude differences remains to be tested directly.

Unanticipated patterns. Two findings were not anticipated by the working framework. The statistical indistinguishability of France, Spain, and Italy at the country fixed-effects level may reflect a shared Romance-language journalistic tradition or a common Continental science journalism culture, but may also partly reflect the lower precision of the Spanish and Italian estimates given their thinner per-country-quarter article volumes (28–35 articles vs. 120–142 in the Anglo-American sample). The dual COVID-19 signature—simultaneous sentiment improvement and risk-frame intensification—suggests that the pandemic produced a polarization of frames rather than a unidirectional shift, consistent with editorial responses to surging audience demand combined with retained gatekeeping responsibility. Both observations generate specific hypotheses for future cross-national research rather than confirmed mechanistic conclusions.

### 5.3 Broader implications

The implications of these findings extend across three scholarly and applied domains. We frame them throughout as hypotheses informed by descriptive cross-national evidence, given that the present study analyzes media content rather than audience attitudes or policy outcomes directly.

For media and communication scholarship. Methodologically, this study demonstrates that panel data econometric approaches can be productively applied to comparative health framing research, enabling the decomposition of cross-national variance into country-level structural components and time-varying shock components in a way that case-comparative designs cannot. At the same time, the six-country panel illustrates the inferential limits of this approach when cluster numbers are small, and underscores the necessity of cluster-robust corrections (such as wild cluster bootstrap) for credible inference in this design class. Substantively, the persistence of country fixed effects across a fifteen-year window provides empirical grounding for treating national media systems as durable framing environments rather than as proximate reflections of contemporaneous events.

For health communication practice. If the relationship between media framing and audience health attitudes documented in prior experimental work (14, 15, 31) extends to the TCM context, our results suggest a testable hypothesis that the risk-dominant Anglo-American media environment may constitute a structural barrier to integrative medicine acceptance, while the more scientifically receptive German and French environments may constitute permissive conditions. This hypothesis is consistent with documented cross-national differences in CAM utilization rates but cannot be tested within the present design; audience-level survey or experimental evidence linking frame exposure to TCM-related health beliefs and behaviors would be required to confirm the inferential chain. We therefore advance this implication as a direction for testing rather than a finding to be acted upon.

For integrative medicine governance. One finding has more direct policy relevance: the ICD-11 inclusion was associated with elevated policy frame ratios but not with improved evaluative tone toward TCM. This descriptive pattern is consistent with the proposition that international institutional legitimation, while effective at shifting which dimensions of TCM are discussed, is insufficient on its own to alter how positively those dimensions are evaluated. Domestic legislative developments, by contrast, were associated with improvements in both sentiment and policy framing within the corresponding national media environment. These observations, although correlational, suggest that policy advocacy strategies relying solely on international institutional signals may face limits in shifting media tone, and that complementary domestic regulatory and evidence-based communication efforts may be required—though the specific causal pathways linking institutional signals to media tone are not identified within the present design.

### 5.4 Future research directions

First, building on the XLM-RoBERTa triangulation analysis on the English corpus reported in Section 4.4 Panel G, the cross-lingual measurement design—VADER for English with human verification, full human coding for non-English—should be replaced in subsequent work by a uniformly fine-tuned multilingual transformer model (e.g., XLM-RoBERTa) applied to all language corpora, allowing observed Anglo-American versus Continental European sentiment differences to be cleanly separated from instrument effects. Second, the inferential chain from media framing to health attitudes and policy support, which the present study can only address by reference to prior experimental literature, should be tested directly by linking frame-environment indicators to audience-level surveys of TCM-related health beliefs, or by experimental designs manipulating frame exposure. Third, the geographic and platform scope should be extended: incorporating digital-native and social media discourse would test whether the country-level patterns documented for elite print media generalize to broader audience environments, and extending to non-Western and non-Anglo-European national contexts would test the boundary conditions of the cross-national heterogeneity reported here. Within the Western European sample, the apparent Romance-language cluster homogeneity warrants targeted replication with larger per-country article samples to distinguish genuine convergence from low-power statistical indistinguishability.

## 6 Limitations

### 6.1 Sampling

The corpus is restricted to elite print and online outlets in the six sampled countries. Tabloid press, regional outlets, and digital-native and social media discourse are not represented. Generalizability to these environments—which may carry distinct framing logics, audience compositions, and editorial gatekeeping standards—remains an open question. Within the included outlets, the panel is also substantially unbalanced in raw article volume across countries, a feature whose statistical implications are addressed below under measurement precision.

### 6.2 Coding comparability

Cross-lingual coding introduces the possibility of translation and instrument artifacts, as reflected in the lower Cohen’s *κ* for Italian-language articles (κ = 0.77 vs. 0.83 for German). More substantively, the hybrid design—VADER for English articles with human verification, full human coding for non-English—applies non-equivalent measurement instruments across language strata. The XLM-RoBERTa triangulation analysis reported in Section 4.4 Panel G addresses one form of this concern by demonstrating that the English-corpus sentiment estimates are robust to the choice of automated instrument; however, the residual non-equivalence between machine-coded English and human-coded non-English remains a constraint of the present design, and full multilingual application of a single transformer model across all six language corpora is identified as a priority for future work (Section 5.4).

### 6.3 Measurement precision

With only six country clusters, asymptotic cluster-robust standard errors are likely downward-biased; wild cluster bootstrap *p*-values should therefore be regarded as the more reliable inferential basis throughout. The panel is also unbalanced in underlying article density: the United States and United Kingdom contribute approximately 120–142 articles per country-quarter, compared with 28–35 for Spain and Italy. Quarterly frame and sentiment estimates for Southern European countries therefore carry higher sampling variance, and cross-national comparisons involving these two countries should be interpreted with corresponding caution.

### 6.4 Causal interpretation

The inferential chain from media framing to public health beliefs and behaviors is not directly tested in the present design, which analyzes media content alone. The public health and communication-strategy implications discussed in Section 5.3 are therefore inferential extensions rather than demonstrated effects. For time-invariant country characteristics—most consequentially, healthcare system type—the Hausman rejection of random effects consistency means these coefficients are identified only under the RE/Mundlak framework and should be read as conditional associations rather than causally identified parameters. With six country clusters, all claims about time-invariant country-level associations are inherently constrained in inferential precision.

## 7 Conclusion

Building on recent cross-national and computational work (10, 11), this study provides one of the first balanced panel data analyses using two-way fixed effects regression of Western news media framing of Traditional Chinese Medicine, documenting substantial and persistent cross-national heterogeneity across six countries over a fifteen-year period. National TCM regulatory developments and major public health events—most notably the COVID-19 pandemic and the WHO’s ICD-11 inclusion of TCM—are each associated with consistent conditional shifts in frame type distribution and sentiment orientation across specifications, though readers should note that inference is based on a six-country panel with a single cluster unit per country and substantial cross-national disparity in article density (with Southern European comparisons particularly subject to higher sampling variance), and statistical precision is inherently constrained at this sample size. The association between NHS-type healthcare system context and reduced sentiment orientation is directionally consistent across RE and Mundlak specifications, but faces a compounded identification challenge: it is estimated under a random effects framework whose consistency assumption is rejected by the Hausman test, and it does not survive wild cluster bootstrap correction (bootstrap *p* = 0.062). This finding should therefore be treated as a preliminary directional signal warranting further investigation rather than a confirmed parameter estimate. The most pronounced cross-national divergence runs between Germany’s receptive scientific framing environment and the United Kingdom’s risk-dominant baseline. These findings generate hypotheses for public health communication: the cross-national heterogeneity of Western media environments suggests that country-specific rather than uniform international TCM communication strategies may be more appropriate, and the limited tone shift following ICD-11 inclusion suggests that institutional legitimation signals alone may be insufficient to shift the evaluative tone of coverage in risk-skeptical media contexts. Both implications, however, require audience-level testing to be confirmed as actionable strategy recommendations. Future research should extend this framework to digital and social media platforms, link media frame indicators to individual-level health belief measures, and broaden the geographic scope beyond the Western European and North American contexts examined here.

## Data Availability

The original contributions presented in the study are included in the article/Supplementary material, further inquiries can be directed to the corresponding author.

## Footnotes

1
https://www.mynews.es
