# Supplementary material for: Media framing of traditional Chinese medicine and its public health implications: a cross-national panel analysis of news coverage in the US, UK, and Europe (2010–2024)
Source: Front Public Health. 2026 Jun 12;14:1826131. doi: 10.3389/fpubh.2026.1826131 (PMC13303672; doi:10.3389/fpubh.2026.1826131)
Supplement: Supplementary file 1 [file Table_1.docx]

**Appendix 1. Coding Manual**

**1.1 General Coding Instructions**

Each article retrieved from the media corpus is coded independently along three analytical dimensions: frame type, sentiment orientation, and article volume weight. All coding decisions are made at the level of the individual article, based on the article's overall narrative thrust rather than isolated sentences or paragraphs. Coders should read each article in full before assigning any codes. When an article's dominant frame or sentiment orientation is ambiguous, coders should record the code that best characterizes the preponderant evaluative stance across the article as a whole.

For English-language articles, a validated machine-assisted sentiment analysis pipeline is applied as the first-pass procedure, with a random 20% subsample subject to human verification. For all non-English articles, human coding by trained bilingual research assistants is the primary procedure. All coders complete a standardized training protocol using a set of 30 calibration articles per language before proceeding to the main corpus. Inter-rater reliability is assessed using Cohen's κ on a random 15% subsample of each language-specific corpus, with a minimum threshold of κ ≥ 0.75 required before full coding proceeds.

**1.2 Inclusion and Exclusion Criteria**

Inclusion criteria: An article is included in the corpus if it meets all of the following conditions:

1. Published within the observation window (January 2010 – December 2024)
2. Published in one of the twelve designated outlets listed in Table 1
3. Contains at least one of the designated keyword terms in the headline, subheadline, or lead paragraph (first three paragraphs)
4. Primarily concerns TCM as a health-related topic (as opposed to purely cultural, culinary, or tourism-related mentions)

Exclusion criteria: An article is excluded if it meets any of the following conditions:

1. Word count below 150 words (news briefs, captions, or listings)
2. TCM mentioned only incidentally as a single subordinate clause within an article primarily concerning another topic
3. Duplicate publication (syndicated reprints are counted once, attributed to the original outlet)
4. Letters to the editor or reader-submitted content

Keyword protocol: Articles are retrieved using the following search terms and their language equivalents:

| **English** | **German** | **French** | **Spanish** | **Italian** |
| --- | --- | --- | --- | --- |
| Traditional Chinese Medicine | Traditionelle Chinesische Medizin | Médecine traditionnelle chinoise | Medicina tradicional china | Medicina tradizionale cinese |
| TCM | TCM | MTC | MTC | MTC |
| Acupuncture | Akupunktur | Acupuncture | Acupuntura | Agopuntura |
| Herbal medicine | Kräutermedizin | Phytothérapie chinoise | Medicina herbal china | Erboristeria cinese |
| Chinese herbal remedy | Chinesische Kräuterheilkunde | Remède à base de plantes chinoises | Remedio herbal chino | Rimedio erboristico cinese |

**1.3 Frame Type Coding**

Frame type is classified into four non-mutually-exclusive categories. An article may be assigned more than one frame code if multiple frames are substantively present. Coders record the presence (1) or absence (0) of each frame type independently.

Frame 1: Scientific/Efficacy Frame

*Definition:* The article's primary evaluative focus is on the clinical evidence base for TCM, including reference to randomized controlled trials, systematic reviews, meta-analyses, expert medical opinion, or lack of scientific validation. The article treats empirical evidence as the primary criterion for evaluating TCM's legitimacy.

*Indicator expressions:* "clinical trial," "evidence-based," "randomized," "Cochrane review," "no scientific evidence," "proven effective," "lacks evidence," "placebo-controlled."

*Coding rule:* Code as 1 if at least one substantive paragraph addresses the empirical evidence base for TCM efficacy or safety in clinical terms. Code as 0 if clinical evidence is mentioned only in passing without constituting a primary evaluative lens.

Frame 2: Risk/Safety Frame

*Definition:* The article foregrounds potential harms, adverse effects, drug interactions, contamination, regulatory failures, or consumer safety risks associated with TCM products or practices.

*Indicator expressions:* "side effects," "adverse reaction," "toxic," "contaminated," "unregulated," "dangerous," "health risk," "warning," "recall," "poisoning."

*Coding rule:* Code as 1 if the article devotes substantive attention to safety concerns, regulatory deficiencies, or documented harms as a primary narrative focus. Code as 0 if safety concerns are acknowledged but subordinate to other frames.

Frame 3: Cultural/Political Frame

*Definition:* The article contextualizes TCM primarily within narratives of Chinese national identity, cultural heritage, geopolitical relations, soft power diplomacy, or ideological contestation between Eastern and Western medical traditions.

*Indicator expressions:* "Chinese culture," "soft power," "diplomatic," "Belt and Road," "cultural heritage," "geopolitical," "China's influence," "Western vs. Eastern medicine."

*Coding rule:* Code as 1 if the article's primary framing situates TCM as a cultural, diplomatic, or geopolitical phenomenon rather than primarily as a clinical or regulatory matter. Code as 0 if cultural references are incidental to a primarily clinical or policy-oriented narrative.

Frame 4: Policy Frame

*Definition:* The article focuses primarily on regulatory developments, insurance reimbursement decisions, legislative proposals, institutional integration of TCM into national healthcare systems, or professional licensing and standardization.

*Indicator expressions:* "regulation," "legalization," "reimbursement," "insurance coverage," "licensing," "standardization," "integrate into healthcare," "policy," "legislation," "statutory."

*Coding rule:* Code as 1 if the article devotes substantive attention to institutional, regulatory, or policy dimensions of TCM governance. Code as 0 if policy references are incidental.

**1.4 Sentiment Orientation Coding**

Sentiment orientation is coded as a single mutually exclusive category reflecting the article's overall evaluative stance toward TCM.

| **Code** | **Label** | **Definition** |
| --- | --- | --- |
| 1 | Positive | The article's overall narrative evaluates TCM favorably, emphasizing benefits, efficacy, cultural value, or policy progress. Critical elements may be present but are subordinate to the dominant positive orientation. |
| 0 | Neutral | The article presents TCM in a balanced or purely descriptive manner without a discernible evaluative stance, or presents positive and negative elements in roughly equal proportion. |
| −1 | Negative | The article's overall narrative evaluates TCM unfavorably, emphasizing risks, lack of evidence, regulatory failures, or cultural suspicion. Positive elements may be present but are subordinate to the dominant negative orientation. |

*Decision rule for ambiguous cases:* When coders are uncertain between Positive and Neutral, or between Negative and Neutral, the Neutral code is assigned. When uncertain between Positive and Negative, coders flag the article for adjudication by a third coder.

**1.5 Article Volume Weight**

Each article is assigned a volume weight based on the estimated national audience reach of its outlet, derived from the most recent available circulation or unique monthly visitor figures for the corresponding year. Weights are normalized within each country-year cell such that the sum of weights across all outlets in a given country-year equals 1.0. Unweighted article counts are retained as an alternative variable for robustness testing.

**1.6 Reliability Assessment Protocol**

Two distinct reliability assessment procedures are applied, corresponding to the two coding modalities used in the project. For English-language articles, which are coded using the VADER machine-assisted sentiment pipeline as the primary procedure, a random 20% subsample is independently coded by a human coder; machine–human agreement is quantified using Cohen’s κ and serves as the validity check for the automated coding. For non-English articles, which are coded by trained bilingual human research assistants, inter-rater reliability between two independent coders is assessed using Cohen’s κ on a randomly drawn 15% subsample of each language-specific corpus. These two procedures are therefore independent and address different sources of coding error: the 20% English subsample assesses machine validity, while the 15% non-English subsample assesses human coder consistency. In both cases, coders are blind to each other’s assignments, reliability is assessed separately for each coding dimension (frame type per frame, sentiment orientation), and discrepancies are resolved through discussion and consensus prior to final dataset construction. Both reliability assessments are conducted prior to full coding of the remaining corpus.

**Appendix 2. Complete Media Sample**

**2.1 Sample Overview by Country**

| **Country** | **Healthcare System Type** | **No. of Outlets** | **Language** | **Total Articles (est.)** |
| --- | --- | --- | --- | --- |
| United States | Market-oriented | 3 | English | ~4,200 |
| United Kingdom | NHS | 3 | English | ~3,800 |
| Germany | Social Health Insurance | 2 | German | ~2,100 |
| France | Social Health Insurance | 2 | French | ~1,900 |
| Spain | Social Health Insurance | 1 | Spanish | ~900 |
| Italy | Social Health Insurance | 1 | Italian | ~850 |
| Total |  | 12 |  | ~13,750 |

*Note: Article counts are approximate pre-screening totals based on raw keyword retrieval returns before application of the inclusion/exclusion criteria described in Appendix 1.2. These figures will therefore exceed the final analytical sample counts. Outlet-level post-screening counts are reported in Appendix 2.3, and country-level final totals are reported in Appendix Table A1. Readers should note that the article counts cited in the main text (e.g., United States: 3,214; United Kingdom: 2,791 in Section 5.2) correspond to the post-screening final analytical corpus (Appendix 2.3 and Table A1), not to the pre-screening raw retrieval totals reported in this section.*

**2.2 Outlet-Level Details**

| **Country** | **Outlet** | **Outlet Type** | **Retrieval Database** | **Language** | **Estimated National Reach** | **Circulation / UMV Basis** |
| --- | --- | --- | --- | --- | --- | --- |
| US | The New York Times | National broadsheet | Factiva / LexisNexis | English | High | Print + digital subscribers |
| US | The Washington Post | National broadsheet | Factiva / LexisNexis | English | High | Print + digital subscribers |
| US | The Wall Street Journal | National broadsheet | Factiva / LexisNexis | English | High | Print + digital subscribers |
| UK | The Guardian | National broadsheet | Factiva | English | High | Unique monthly visitors |
| UK | The Times | National broadsheet | Factiva | English | High | Print + digital subscribers |
| UK | BBC Online News | National online outlet | Factiva | English | Very High | Unique monthly visitors |
| DE | Der Spiegel | National news magazine | Genios | German | High | Print + digital subscribers |
| DE | Frankfurter Allgemeine Zeitung | National broadsheet | Genios | German | High | Print circulation |
| FR | Le Monde | National broadsheet | Europresse | French | High | Print + digital subscribers |
| FR | Le Figaro | National broadsheet | Europresse | French | High | Print + digital subscribers |
| ES | El País | National broadsheet | MyNews (mynews.es) | Spanish | High | Print + digital subscribers |
| IT | La Repubblica | National broadsheet | Factiva | Italian | High | Print + digital subscribers |

**2.3 Keyword Retrieval Results by Outlet**

Note: All article counts in this table are approximate figures (~) derived from raw keyword retrieval logs prior to the application of full inclusion/exclusion screening. They are intentionally rounded to the nearest 10 to reflect retrieval-stage uncertainty. The corresponding exact post-screening counts for each country are reported in Table A1 below; outlet-level figures in this table aggregate to the country totals shown there (e.g., US outlets: ~1,210 + ~1,090 + ~900 = ~3,200 ≈ 3,214 exact; UK outlets: ~1,170 + ~900 + ~730 = ~2,800 ≈ 2,791 exact). Discrepancies of ±14 or fewer articles between the summed approximations and Table A1 exact counts are within expected rounding tolerance and do not indicate data errors.

| **Country** | **Outlet** | **Raw Hits (pre-screening)** | **Excluded (criteria)** | **Final Analytic N** |
| --- | --- | --- | --- | --- |
| US | The New York Times | ~1,520 | ~310 | ~1,210 |
| US | The Washington Post | ~1,380 | ~290 | ~1,090 |
| US | The Wall Street Journal | ~1,300 | ~400 | ~900 |
| UK | The Guardian | ~1,450 | ~280 | ~1,170 |
| UK | The Times | ~1,210 | ~310 | ~900 |
| UK | BBC Online News | ~1,140 | ~410 | ~730 |
| DE | Der Spiegel | ~1,060 | ~250 | ~810 |
| DE | Frankfurter Allgemeine Zeitung | ~1,040 | ~250 | ~790 |
| FR | Le Monde | ~980 | ~240 | ~740 |
| FR | Le Figaro | ~920 | ~230 | ~690 |
| ES | El País | ~900 | ~260 | ~640 |
| IT | La Repubblica | ~850 | ~270 | ~580 |
| Total |  | ~13,750 | ~3,500 | ~10,250 |

**2.4 Appendix Table A1. Corpus Article Counts and Pre-COVID Growth Rates by Country**

Table A1 reports exact article counts by country and the pre-COVID average annual growth rates in quarterly coverage volume (2010 Q1 – 2019 Q4), estimated via log-linear regression of log(quarterly count) on a linear time trend. Counts are post-exclusion final corpus totals. Growth rates are compound annual growth rates derived from the regression slope coefficient (β̂ × 4 to annualise from quarterly estimates). Mean articles per country-quarter are computed as total articles ÷ 60 quarters and rounded to one decimal place using conventional half-up rounding throughout (e.g., Germany: 1,587 ÷ 60 = 26.45 → 26.5; France: 1,437 ÷ 60 = 23.95 → 24.0).

| **Country** | **N outlets** | **Total articles (N)** | **Mean articles / country-quarter** | **Pre-COVID CAGR (2010–2019)** |
| --- | --- | --- | --- | --- |
| United States | 3 | 3,214 | 53.6 | 4.2% |
| United Kingdom | 3 | 2,791 | 46.5 | 3.8% |
| Germany | 2 | 1,587 | 26.5 | 8.3% |
| France | 2 | 1,437 | 24.0 | 7.1% |
| Spain | 1 | 643 | 10.7 | 5.6% |
| Italy | 1 | 578 | 9.6 | 6.1% |
| **Total** | **12** | **10,250** | **28.5** | **—** |

**2.5 Appendix Table A2. Wild Cluster Bootstrap p-Values for Primary Coefficient Estimates (TWFE)**

The following table reports asymptotic cluster-robust p-values alongside wild cluster bootstrap p-values for all primary coefficient estimates. Bootstrap p-values are based on 999 replications with Rademacher weights, implemented via the Stata boottest command (Roodman et al., 2019). Given the six-country panel, asymptotic cluster-robust p-values may be downward-biased; bootstrap p-values provide finite-sample corrections. Qualitative conclusions are substantially unchanged across primary findings, with one exception: the NHS System effect on the sentiment index does not survive wild cluster bootstrap correction (asymptotic p = 0.029; bootstrap p = 0.062), and should be interpreted as suggestive rather than definitive (see Section 5.4, Limitation 6).

| **Variable** | **Sentiment Index (asymp. p / boot. p)** | **Risk Frame Ratio (asymp. p / boot. p)** | **Policy Frame Ratio (asymp. p / boot. p)** |
| --- | --- | --- | --- |
| TCM Legislative Status | 0.009 / 0.016 | 0.521 / 0.544 | 0.012 / 0.020 |
| COVID-19 | 0.000 / 0.000 | 0.007 / 0.016 | 0.121 / 0.147 |
| ICD-11 Inclusion | 0.214 / 0.249 | 0.281 / 0.312 | 0.006 / 0.012 |
| NHS System | 0.029 / 0.062 | 0.007 / 0.016 | 0.753 / 0.781 |
| Social Insurance System | 0.548 / 0.581 | 0.021 / 0.047 | 0.004 / 0.012 |
| COVID-19 × NHS System | 0.446 / 0.484 | 0.009 / 0.031 | 0.312 / 0.358 |
| ICD-11 × Social Ins. | 0.312 / 0.351 | 0.644 / 0.672 | 0.086 / 0.109 |

*Note: Each cell reports asymptotic cluster-robust p-value / wild cluster bootstrap p-value. * p < 0.05, ** p < 0.01, *** p < 0.001. Interaction term p-values are derived from the Table 4 interaction model. Wild cluster bootstrap implemented via Stata boottest (38).*
